# Can the Attention Training Technique Reduce Stress in Students? A Controlled Study of Stress Appraisals and Meta-Worry

**DOI:** 10.3389/fpsyg.2019.01532

**Published:** 2019-07-10

**Authors:** Peter Myhr, Timo Hursti, Katarina Emanuelsson, Elina Löfgren, Odin Hjemdal

**Affiliations:** ^1^ MCT-Stockholm, Private Practice, Stockholm, Sweden; ^2^ Department of Psychology, Uppsala University, Uppsala, Sweden; ^3^ Department of Psychology, Norwegian University of Science and Technology, Trondheim, Norway; ^4^ Division of Psychiatry, St. Olavs Hospital, Nidaros DPS, Trondheim, Norway

**Keywords:** attention training, stress, meta-worry, S-REF model, experimental study

## Abstract

The present study tested the impact of attention training on cognition; secondary appraisal of perceived stress, and on metacognition; meta-worry in stressed students. Theoretically derived from the Self-Regulatory Executive Function model (S-REF model; Wells and Matthews, 1994a, 1996), the attention training technique (ATT; Wells, 1990) is intended to promote flexible, voluntary external attention and has been shown to reduce symptoms of psychological distress. The present experimental study explored the effects of ATT on cognitive and metacognitive levels of appraisal, namely perceived stress (primary outcome) and meta-worry (secondary outcome). Stressed students were randomized to an experimental ATT group (*n* = 23) or a control group (*n* = 23). The ATT group attended an initial training session followed by 4 weeks of individual (12 min) daily ATT practice. The control group waited for 4 weeks before receiving the intervention. The outcomes were scores on the Perceived Stress Scale 14 (PSS-14) and the Meta-Worry Questionnaire (MWQ) frequency and belief subscales at post study. Both measures decreased significantly following ATT with large pre- to post- effect sizes but there were minimal changes in the control group. The between-group differences were statistically significant. The results add to the literature on the potential effects of ATT by demonstrating effects on the content of cognitive stress appraisals and on meta-worry in an academic setting in a stressed student sample.

## Introduction

In the Self-Regulatory Executive Function model (S-REF; Wells and Matthews, 1994a, 1996), stress reactions are viewed as arising from the activation of a pattern of processing called the cognitive attentional syndrome (CAS) that is marked by self-focused attention and consists of repetitive conceptual processing of negative information (worry, rumination, threat monitoring). In this model more severe stress reactions are linked to a higher level of unhelpful metacognitions, especially those beliefs that thinking cannot be controlled or is harmful (i.e., meta-worry or worry about worrying; Wells, 1994).

One of the first techniques developed to alleviate the CAS was the attention training technique (ATT: Wells, 1990), which involves the practice of specific auditory attention control exercises. The technique shows evidence of effectiveness for a range of anxiety and depression symptoms as reported in systematic reviews (Wells et al., 1997; Papageorgiou and Wells, 1998, 2000; Siegle et al., 2007, 2014; Calkins et al., 2015; Fergus and Bardeen, 2016). Furthermore, there is emerging evidence that the effects are detectable in neurocognitive measures associated with executive control of attention (Knowles et al., 2016).

While the ATT has been applied to clinical states such as anxiety and depression symptoms, the effect of the technique on more general stress-related appraisals has yet to be evaluated. In addition, the impact of ATT on cognition as well as metacognition levels of appraisal in stress remains to be tested. Studies from clinical samples show that the technique can reduce rumination and modify metacognitive beliefs. However, Wells and Matthews (1994a) see the CAS as potentially undermining secondary appraisals of the ability to cope with challenges. Thus, ATT should have an effect on cognitive appraisals of ability to cope with stress as well as impacting on metacognition (i.e., meta-worry). The current study set out to test whether the ATT can work in a stressed sample by impacting on cognitive and metacognitive appraisals.

The transactional theory of stress (Lazarus and Folkman, 1984) views stressful situations as those that are appraised as taxing or exceeding the individual’s resources to cope. This process is closely tied to primary and secondary appraisals. Primary appraisals concern the evaluation of events in the terms of initial threat or challenges while secondary appraisal refers to what can be done to cope and the likely success of responses. The S-REF model (Wells and Matthews, 1994a, 1996) on the other hand links stress to the activation of a syndrome of cyclical thinking in the form of worry and rumination coupled with diminished perceptions of metacognitive control over thinking. Wells and Matthews have argued that the deleterious effects of the syndrome are most likely to be observed in situations that are cognitively demanding such as those that are ambiguous or contain uncertainty because they require more cognitive resources that are depleted by the syndrome. Consistent with this idea, Wells and Matthews (1994b) demonstrated a negative association between self-focused attention (a marker for the CAS) and emotion-focused and problem-focused coping only in mixed controllability situations.

In the present study, we set out to test the effects of an ATT intervention on the mechanisms of stress implicated in the transactional theory and the S-REF model. To reach this aim, we therefore assessed stress appraisals consistent with Lazarus and Folkman’s model as a primary outcome and metacognitive appraisals in the form of meta-worry consistent with the S-REF as a secondary outcome. Meta-worry was first identified by Wells (1994) as a process of worrying about worry and is a dysfunctional metacognitive appraisal process. It consists of appraising worry as uncontrollable and dangerous and is thought to be closely associated with underlying metacognitive beliefs. Meta-worry is an important process contributing to the CAS in psychopathology including generalized anxiety disorder (Wells, 1995, 2005).

### Aim

In the present study, we tested the impact of the ATT on stress-related appraisals and meta-worry in stressed students. We aimed to address the question: can the ATT reduce stress appraisals and meta-worry?

## Materials and Methods

### Design

The study uses a randomized controlled experimental design comparing the ATT with a wait-list control group. There were pre- and post-treatment assessments regarding outcome measures.

### Participants

The majority of the participants were students within the faculty of social sciences. The inclusion criterion was intended to be as broad as possible. However, to be eligible for the study, students had to have a 75% or higher course load. Part-time students with course load lower than 75% were not included. In addition, students had to self-identify as currently “stressed.” Participation required attending a 45-min training session and then undertaking the ATT 12 min daily for 4 weeks. The study used a convenience sample. Participation was voluntary, and compensation was not typical apart from three participants who received university credits as part of a psychology class at Uppsala University.

The initial sample consisted of 48 participants of whom 34 (71%) identified themselves as women and 13 (27%) as men, and one (2%) had a different gender identity. The participants ranged from 19 to 43 years of age (*M* = 25.4, SD = 5.2).

#### Drop-out

Post-intervention data were collected on 46 of the original 48 participants. One participant withdrew because of limited availability of time and the second for unknown reasons. The dropout rate was 4.17%.

### Material

*Perceived Stress Scale 14 (PSS-14)* was developed by Cohen et al. (1983). It measures the extent to which life situations are evaluated as stressful; thus, it’s items tap primary and secondary appraisals according to the transactional stress model. It consists of seven positive and seven negative items all measured on a 5-point Likert scale from 0 (*never*) to 4 (*very often*). An example positive item is: “In the last month, how often have you dealt successfully with irritating life hassles?” An example negative item is: “In the last month, how often have you felt nervous and stressed?” It has shown good psychometric properties (Lee, 2012). It has been tested in Sweden and has good psychometric properties (Cronbach *α* = 0.84, *α* = 0.90) and indications of validity has been shown as it differentiates between a sample with stress disorder and other samples, as well as predicting sensitivity toward change as significant changes were identified from pre to post in a work rehabilitation intervention (Eklund et al., 2014).

*Meta-Worry Questionnaire (MWQ)* measures the level of meta-worry (Wells, 2005). The response format measures two aspects of meta-worry: the frequency of meta-worry (frequency scale) and the respective belief in the meta-worry (belief-scale). The frequency of meta-worry contains seven statements, for example: “When I worry, I think: I’m abnormal for worrying.” The belief in the same meta-worry thought is measured by responding on a scale from 0 (*I do not believe this thought at all*) to 100 (*I am completely convinced this thought is true*). The psychometric properties are good (for frequency Cronbach *α* = 0.88 and for belief scale *α* = 0.95) and validity has been demonstrated as patients with generalized anxiety disorder score significantly higher than somatic anxiety and no-anxiety groups (Wells, 2005).

### Procedure

Participants were recruited following provision of information about the study that was given in classes at Uppsala University. Information was also given on bulletin boards at Uppsala University, Swedish University of Agricultural Sciences in Uppsala, at the student union offices, students’ health services in Uppsala, as well as shared on social media. Those who volunteered to participate were contacted by phone for further information and settling of specific times for ATT training. All communication and questionnaires were in Swedish, with the exception of the original English ATT soundtrack which was used both in the introduction group session and subsequent individual ATT training sessions.

Two students were responsible for conducting the 45-min introductory group sessions with a maximum of five participants at a time. The students were supervised by an MCT-I certified therapist. The session started with a few minutes of psychoeducation to illustrate how thoughts and appraisals (primary and secondary) may influence perceived stress levels in accordance with the transactional theory of stress. This was followed by an introduction to the CAS and the original rational for the ATT, a practice session of the ATT followed.

ATT involves instructing individuals to focus on external sounds and specific spatial locations. The training consists of three parts focusing on (1) selective attention, (2) rapid change of attention, and (3) divided attention (Wells, 2009). We used a 12-min pre-recorded soundtrack in the implementation of practice following the procedure described by Wells (Wells, 1990). After having completed the ATT, participants were encouraged to ask questions to clarify the use of the ATT.

#### Conditions

*The ATT group* participants were assessed prior to the training and after 4 weeks of training. After the introductory training session, the participants were instructed to perform ATT daily during the following 4-week period. To encourage adherence participants were asked to register the date and time of their individual training, and they were encouraged to continue training even if they missed a day or two. Frequency of individual training was not collected. Two weeks into the ATT training, the participants received an e-mail reminder to continue ATT training. The participants were encouraged not to engage in other self-help activities targeting stress.

*Wait-list* participants were assessed prior and after the 4 weeks waiting period. They were informed that their training period would be introduced after the post-waiting assessment. During their waiting period they were encouraged to live their lives normally until the training would start. After their 4 week waiting period, their training was identical to the one received by the ATT group.

#### Data Collection

The pre- and post-treatment assessments were done *via* a website generated using surveymonkey.com. After the recruitment, participants received an e-mail with a link to the study website where they were provided with further information, after which they provided informed consent before proceeding to the questionnaires. Questionnaires included demographic questions (i.e., gender and age), the PSS-14, and the MWQ. After 4 weeks, participants received a new web link *via* e-mail leading to post-treatment assessment, which included the PSS-14 and MWQ. Filling out the questionnaires took approximately 10 min.

#### Randomization

After inclusion, participants were randomized to either the ATT or wait-list. Randomization was undertaken using www.random.org matching on gender identity. Each group consisted of 24 participants. In the ATT group, the gender identity was 17 women and seven men, and in the wait-list condition 17 women, six men, and one prefering not to disclose gender identity.

### Ethics

The procedures for data collection were performed in accordance with the Declaration of Helsinki, as well as the guidelines for professional conduct of clinical psychologist in the Nordic countries. All participants received a written informed consent in addition to the oral information. Participants were informed about the study, that participation was voluntary and confidential, and they had the right to withdraw from the study without giving any reason or it having consequences. All data were stored anonymously. It was specified in the given information that if participants needed further assistance during or after the project, they could contact the two clinical psychologists involved in the project.

The present study was part of the students’ thesis, and the guidelines for student projects at Uppsala University were followed. The study was ethically reviewed and accepted by the Department of Psychology, Uppsala University.

### Statistical Analyses

Statistical analyses were undertaken using SPSS version 23. A Shapiro-Wilks normality test indicated that the results were normally distributed. Change scores for the PSS-14 were established by subtracting post-scores from pre-scores. Within-group effect sizes were estimated using Cohen’s *d* where 0.2 indicates small, 0.5 medium, and 0.8 large effect. In the ANOVA, effect sizes were estimated using eta squared (*η^2^*), where 0.01 indicates small, 0.059 medium, and 0.138 large effect (Clark-Carter, 2010). Changes in PSS-14 and MWQ scores were explored using a mixed two-way ANOVA, followed up with dependent t-tests. The associations between perceived stress and meta-worry and if participants with higher levels of meta-worry experienced ATT as particularly helpful were explored using correlations.

### Results

#### Changes in Perceived Stress

A mixed two-way ANOVA was conducted using the PSS-14 as a dependent variable. The independent variables were time and group. Time was pre- and post assessment, and group was either ATT or wait-list. The analyses showed a significant main effect for time, *F*(1, 44) = 23.48, *p* < 0.001, with a large effect size of *η^2^* = 0.53. There was no significant main effect for group *F*(1, 44) = 0.24, *p* = 0.63. There was a significant interaction effect (time × group) *F*(1, 44) = 12.43, *p* = 0.001, with a large effect size of *η^2^* = 0.28. The level of perceived stress among stressed university students was significantly reduced after participating in the ATT, in comparison to the wait-list control group.

Further analyses using follow-up t-tests confirmed a significant reduction in PSS-14 scores in the ATT group *t*(22) = 5.52, *p* < 0.001, with a large effect size but the wait-list group did not show a significant reduction over time *t*(22) = 1.01, *p* = 0.32. Mean values and effect sizes (Cohen’s *d*) are reported in Table 1.

**Table 1:** Mean scores, standard deviations, and Cohen’s d for PSS-14, MWQ-F, and MWQ-B for participants in the ATT condition (ATT) and the wait-list (WL).

Instrument	Group	n	Pre M (SD)	Post M (SD)	*d* ES
PSS-14	ATT	23	32.35 (5.25)	24.35 (6.02)	1.18
	WL	23	29.78 (7.98)	28.52 (6.32)	0.18
MWQ-F	ATT	23	15.70 (3.83)	13.39 (3.03)	0.67
	WL	23	15.17 (4.30)	15.04 (4.27)	0.03
MWQ-B	ATT	23	370.91 (152.36)	228.09 (191.61)	0.83
	WL	23	312.91 (154.85)	273.30 (186.08)	0.23

*ATT, attention training; WL, wait-list; PSS-14, perceived stress scale 14; MWQ-F, meta-worry questionniare - frequency; MWQ-B, meta-worry questionniare – belief*.

#### Changes in Meta-Worry

To explore changes in frequency of meta-worry, a mixed two-way ANOVA was undertaken. There was a main effect for time, *F*(1, 44) = 8.89, *p* = 0.005, with a large effect size *η^2^* = 0.20, and a significant interaction effect (time × group), *F*(1, 44) = 7.17, *p* = 0.01, also with a large effect size *η^2^* = 0.16. There was no significant main effect for group *F*(1, 44) = 0.27, *p* = 0.60. *T*-tests indicated significant reduction from pre- to post-scores in the ATT group for MWQ-F, *t*(22) = 4.17, *p* < 0.000, the effect size was medium. For the wait-list group, there was no significant reduction for MWQ-F, *t*(22) = 0.21, *p* = 0.84. Mean scores and effect size (Cohen’s *d*) are reported in Table 1.

To explore changes in beliefs in meta-worry (MWQ-B), a similar mixed two-way ANOVA was conducted. Significant main effects were found for time, *F*(1, 44) = 11.49, *p* = 0.001, with a large effect size of *η^2^* = 0.26. There were no significant interaction effects (time × group), *F*(1, 44) = 3.68, *p* = 0.06, neither was there a significant main effect for group, *F*(1, 44) = 0.02, *p* = 0.88. Therefore, beliefs decreased overall in both groups but there was no differential effect observed.

## Discussion

We found an effect of the ATT intervention on levels of perceived stress in university students. The intervention led to greater reductions in stress levels compared with a no-treatment waiting period. We also demonstrated an effect in reducing meta-worry frequency scores. However, the effect on meta-worry belief was non-significant. The results are consistent with an ameliorative effect of ATT on hypothesized mechanisms of stress in stressed students. The reason for a lack of an effect on meta-worry belief levels is unclear; this may be due to the ATT not being effective on this dimension or lack of power to detect such an effect given the small sample size of the study. It should be noted that the initial scores on this dimension were low with high variability and this may have contributed to the size of the effect observed. The within-group effect sizes show a large effect for the perceived stress and meta-worry outcomes. Individuals were included based on their own evaluation of personal stress levels. The mean score of 31.07 was well above a Swedish non-clinical population (Eklund et al., 2014). For women with stress-related disorders, the mean score of 30.0 has been suggested.

The use of a control group that waited for the length of time over which the ATT group received the intervention controls for the passage of time and spontaneous recovery from stress, but it does not control for the non-specific factors involved in delivering an intervention. We cannot be sure that it is the ATT that caused improvement or other factors such as deviations from normal routines caused by practicing the technique, placebo effects, or expectancies of improvement. We aimed to test a more basic question: does it have an effect? This question is useful to clarify before more rigorous studies are planned.

There are other important limitations of the study that should be considered. We delivered ATT in a dose that is below what is normally recommended for clinical samples, where practicing twice a day rather than once is usually advised. Furthermore, we cannot be sure of the actual level of practice that the students adhered to, which is a major limitation. We also combined the ATT with a rationale that described the role of primary and secondary appraisals in stress and this is not part of the usual rationale for ATT that is grounded in the metacognitive model. We cannot ascertain if this hybrid explanation had a detrimental, positive, or no impact on the effectiveness of the intervention. When the ATT is used as a therapeutic intervention it is typically combined with therapist-led guidance and exploration of subjective experiences to re-shape the clients’ maladaptive metacognitions (Wells, 2009).

In conclusion, these findings tentatively add to the research on the effects of ATT by suggesting that it can have an effect on stress responses, which extends the potential utility of ATT to managing stress in non-clinical samples. The results support the further investigation of effects of the technique within this context. Future studies should aim to control non-specific treatment factors and to separate the effects of the attention exercises from the other elements in the package used.

## Ethics Statement

The procedures for data collection were performed in accordance with the Declaration of Helsinki, as well as the guidelines for professional conduct of clinical psychologist in the Nordic countries. All participants received a written informed consent in addition to the oral information. Participants were informed about the study that participation was voluntary and confidential, and they have the right to withdraw from the study without giving any reason or it having consequences. Data were saved anonymized. It was specified in the given information that if participants needed further assistance during or after the project, they could contact two clinical psychologists involved in the project. The present study was part of a student thesis, and the guidelines for student projects at Uppsala University were followed. The study was ethically reviewed and accepted by the Department of Psychology, Uppsala University.

## Author Contributions

PM developed the study and supervised. TH contributed with analyses and supervision. KE and EL ran the experiment. All authors contributed in the writing of the manuscript.

### Conflict of Interest Statement

The authors declare that the research was conducted in the absence of any commercial or financial relationships that could be construed as a potential conflict of interest.
